# *PLEKHA8P1* Promotes Tumor Progression and Indicates Poor Prognosis of Liver Cancer

**DOI:** 10.3390/ijms22147614

**Published:** 2021-07-16

**Authors:** Jiyeon Lee, Ji-Hyun Hwang, Harim Chun, Wonjin Woo, Sekyung Oh, Jungmin Choi, Lark Kyun Kim

**Affiliations:** 1 Severance Biomedical Science Institute, Graduate School of Medical Science, Brain Korea 21 Project, Gangnam Severance Hospital, Yonsei University College of Medicine, Seoul 06273, Korea; iwldus2752@naver.com (J.L.); wwj94@naver.com (W.W.); 2 Interdisciplinary Program of Integrated OMICS for Biomedical Science, The Graduate School, Yonsei University, Seoul 03722, Korea; jiihyun@gmail.com; 3 Department of Biomedical Sciences, Korea University College of Medicine, Seoul 02841, Korea; bgr1663@korea.ac.kr; 4 Department of Medical Science, Catholic Kwandong University College of Medicine, Incheon 22711, Korea; ohskjhmi@gmail.com

**Keywords:** pseudogene, long non-coding RNA, hepatocellular carcinoma, 5-fluorouracil, chemoresistance, *PLEKHA8P1*, *PLEKHA8*

## Abstract

Hepatocellular carcinoma (HCC) records the second-lowest 5-year survival rate despite the avalanche of research into diagnosis and therapy. One of the major obstacles in treatment is chemoresistance to drugs such as 5-fluorouracil (5-FU), making identification and elucidation of chemoresistance regulators highly valuable. As the regulatory landscape grows to encompass non-coding genes such as long non-coding RNAs (lncRNAs), a relatively new class of lncRNA has emerged in the form of pseudogene-derived lncRNAs. Through bioinformatics analyses of the TCGA LIHC dataset, we have systematically identified pseudogenes of prognostic value. Initial experimental validation of selected pseudogene-derived lncRNA (*PLEKHA8P1*) and its parental gene (*PLEKHA8*), a well-studied transport protein in Golgi complex recently implicated as an oncogene in both colorectal and liver cancer, indicates that the pseudogene/parental gene pair promotes tumor progression and that their dysregulated expression levels affect 5-FU-induced chemoresistance in human HCC cell line FT3-7. Our study has thus confirmed cancer-related functions of *PLEKHA8*, and laid the groundwork for identification and validation of oncogenic pseudogene-derived lncRNA that shows potential as a novel therapeutic target in circumventing chemoresistance induced by 5-FU.

## 1. Introduction

Liver cancer is the sixth-most diagnosed cancer and the fourth-most common cause of cancer-related death globally [1], and is estimated to contribute to the deaths of more than one million people in 2030 based on annual projections by the World Health Organization [2]. Hepatocellular carcinoma (HCC) accounts for the majority (90%) of primary liver cancer, presenting itself as a serious global health burden [3]. Clinical management is based mostly on the Barcelona Clinic Liver Cancer (BCLC) staging classification [4]. Potentially curative therapies such as surgical resection, liver transplantation and tumor ablation are recommended for early diagnosis (BCLC stage 0–A) [5], while transarterial chemoembolization (TACE) is the standard-of-care treatment for intermediate stage (BCLC stage B) patients [6]. Patients with advanced stage (BCLC C) diagnosis or those who exhibit disease progression after TACE undergo systemic treatment. Despite established guidelines for a seemingly wide range of therapies, difficulties in early diagnosis and prevalent resistance to chemotherapy-based treatments [4] render most treatments ineffective, and HCC patient survival outcome remains poor, as manifested in its dismal 5-year survival rate (20%), second lowest only to pancreatic cancer [7]. Much attention, therefore, has been devoted to characterizing chemoresistance with the goal of identifying potential therapeutic targets [8].

With the advent of high-throughput sequencing technologies, the role of non-coding RNAs in diseases despite their lack of protein products is unquestionable [9]. Non-coding RNAs are defined arbitrarily by their length—most are more than 200 nucleotides in length and such transcripts are catalogued as long non-coding RNAs (lncRNAs) [10]. Functional studies have revealed pervasive regulatory roles for lncRNAs in various cancers, whereby they participate in virtually every cellular process ranging from cellular proliferation and apoptosis to alternative splicing and chemoresistance [11]. lncRNA classification has also mushroomed over time [12], and the class termed pseudogene-derived RNA transcripts has rapidly gained traction in tumorigenic-related studies [13]. Their relative late emergence into the spotlight can be explained partially by their initial definition—pseudogenes, first coined by Jacq et al. in 1977 [14], were described as non-functional and ‘junk’ genomic loci as they were truncated forms of their protein-coding parental genes [15]. However, as with other lncRNAs classes, growing evidence indicates their pivotal role in initiation and development of human cancer [16] and more so in relation to their parental genes [15,17]. Given their high tissue specificity relative to protein-coding genes [18] and elegantly complex yet precise mode-of-action [19], it is unsurprising that pseudogene-lncRNAs have already been implicated in HCC chemoresistance [16]. As pseudogenes share high sequence homology with their parental genes [20], unraveling the relationship between pseudogene/parental genes and their resulting effect upon gene regulation is clinically relevant, since more precise candidates can be designated as therapeutic targets. The major model mechanism-wise proposed for pseudogene/parental gene pair is the competing endogenous RNA (ceRNA) model, whereby the pair compete for the same microRNA (miRNA) through shared miRNA binding sites [21,22,23]. However, given various challenges such as difficulty in discerning between pseudogenes and their parental genes [20] and the ever-growing pool of pseudogene-lncRNAs [24], there remains much room for continuous investigation of molecules that are involved in HCC chemoresistance.

Drug resistance to chemotherapy is a major obstacle in HCC treatment, and is driven partly by cells that acquire survival advantages, mediated by genes involved in various processes such as cell survival, migration/invasion and epithelial–mesenchymal transition (EMT) [25]. Identification of key genes is thus critical and lncRNAs are excellent candidates, as several studies have demonstrated their regulatory effect on such processes in HCC [26,27,28,29,30].

In this study, we have identified a pseudogene-derived lncRNA (*PLEKHA8P1*) and its corresponding parental gene (*PLEKHA8*), which promotes chemoresistance to 5-fluorouracil (5-FU) in the human HCC cell line FT3-7 (clonal derivative of Huh-7 cells). Though initially characterized as an integral member in membrane trafficking [31,32,33], studies have revealed the tumorigenic role of *PLEKHA8* in various cancers, including HCC by enhancing the Wnt/β-catenin pathway [33,34,35,36]. We have shown that the *PLEKHA8P1*/*PLEKHA8* pair confers an oncogenic role through cell proliferation, migration/invasion and wound healing assays, and that it potentially enhances HCC 5-FU-induced chemoresistance. Our study thus contributes to the fledgling field of the identification and characterization of novel therapeutic pseudogene-lncRNA/parental gene pairs that modulate chemoresistance.

## 2. Results

### 2.1. PLEKHA8P1 Is Up-Regulated in HCC Samples and Predicts Unfavorable Prognosis in Patients from the TCGA-LIHC Dataset

**Figure 1:**
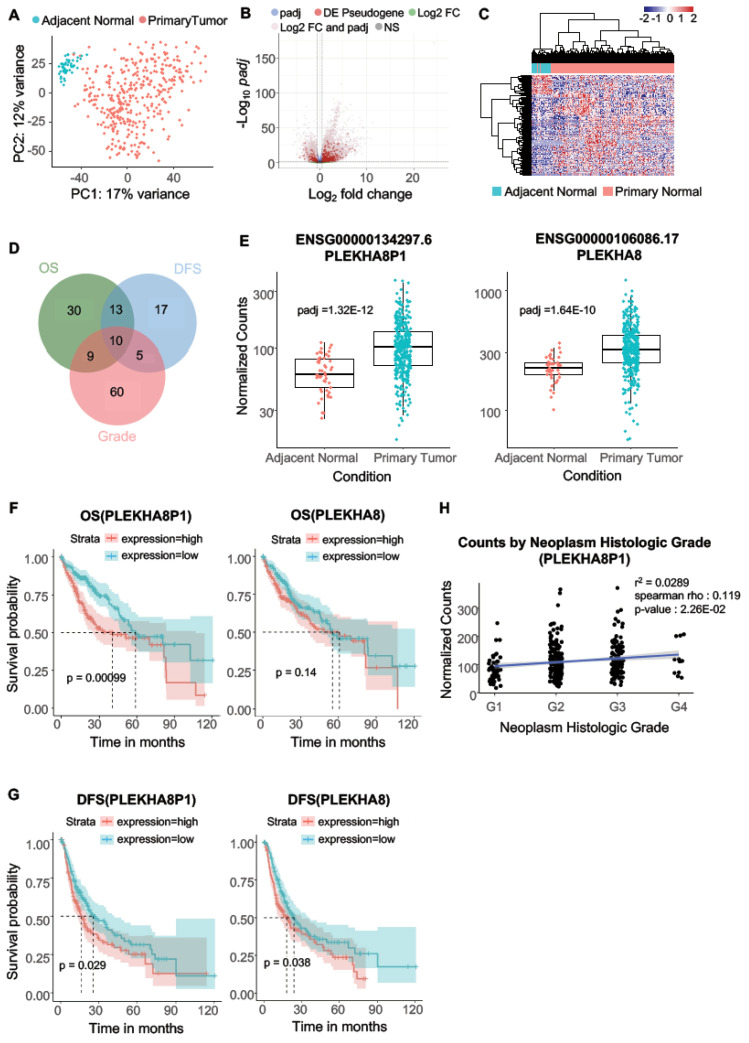
Identification of differentially expressed pseudogene with clinical implications in liver cancer. (**A**) Principal component analysis (PCA) plot of 369 primary liver tumor samples (red) and 49 adjacent normal samples (blue). (**B**) Significantly differentially expressed pseudogenes are marked in red in volcano plot. (**C**) Hierarchical clustering analysis of 465 significantly dysregulated pseudogenes in primary liver tumors (red) and adjacent normal tissues (blue). (**D**) Overlap between differentially expressed pseudogenes based on association with three clinical features (OS, DFS, histologic grade). (**E**) Expression of *PLEKHA8P1* (left panel) and *PLEKHA8* (right panel) in primary liver tumors (red) and adjacent normal tissues (blue). (**F**,**G**) Kaplan–Meier survival curves of *PLEKHA8P1* (left panel) and *PLEKHA8* (right panel) associated with overall survival and disease-free survival. (**H**) Correlation analysis between *PLEKHA8P1* expression level and the neoplasm histologic grade. Blue line is the regression line, and the gray shadow indicates the confidence interval.

To explore dysregulated pseudogenes in human liver cancer, we first downloaded and processed the TCGA LIHC (liver hepatocellular carcinoma) dataset comprising 369 primary liver tumor tissues with 49 adjacent normal tissues (Figure 1A). Out of the listed 2913 pseudogenes with known parent genes (Supplementary Table S1), 465 pseudogenes (Supplementary Table S2) showed significantly altered expression in primary liver tumors (|Log_2_FC| > 0.5, Adjusted *p*-value < 0.05, Figure 1B,C). We further evaluated the correlation between the 465 differentially expressed pseudogenes and histopathological tumor grade in primary liver tumors to assess their association with clinicopathological features, and found 84 genes to be significantly correlated with tumor grade (Spearman *p*-value < 0.05, Supplementary Table S3). Subsequently, we also investigated the prognostic values of the 465 pseudogenes utilizing clinical outcome data by the Kaplan–Meier method. A total of 62 pseudogenes indicated significant association (log rank *p*-value < 0.05, Supplementary Table S3) with overall survival (OS), while 45 pseudogenes showed strong correlation (log rank *p*-value < 0.05, Supplementary Table S3) with disease-free survival (DFS). We sought to derive common pseudogenes that displayed significance in all three analysis sets (Figure 1D, Supplementary Table S3) for stringent candidate selection. Ten such common pseudogenes were retrieved and we focused on the pseudogene-derived lncRNA, Pleckstrin homology domain containing A8 pseudogene 1 (*PLEKHA8P1*), as it was previously reported to predict overall survival and recurrence in renal cell carcinoma [37]. A significantly higher level of *PLEKHA8P1* was observed in primary liver tumors compared with adjacent normal tissues (Figure 1E). To test the relationship of pseudogene and its parental gene, we examined the gene expression of a parent gene, *PLEKHA8* in primary liver tumors. Significant upregulation was found in primary liver tumors, suggesting *PLEKHA8P1* may promote liver cancer progression by up-regulating its parent gene, *PLEKHA8* (Figure 1E). Patients with high expression level of *PLEKHA8P1* trend towards decreased survival rates in both OS and DFS (log rank *p*-value = 0.00099 and 0.029, respectively); however, patients with an elevated level of *PLEKHA8* exhibited poorer prognosis only for DFS (log rank *p*-value = 0.038, Figure 1F,G). Furthermore, expression of *PLEKHA8P1* positively correlated with histological grade of primary liver tumors (Spearman *p*-value = 0.0226, Figure 1H). Taken together, these findings indicate that *PLEKHA8P1* and its parent gene, *PLEKHA8*, promote tumorigenesis in liver cancer and *PLEKHA8P1* demonstrates a better prognostic value than its parental gene.

### 2.2. Selection of Antisense Oligonucleotide (ASOs) for Precise Knock-Down of PLEKHA8P1 in HCC Cell Line

**Figure 2:**
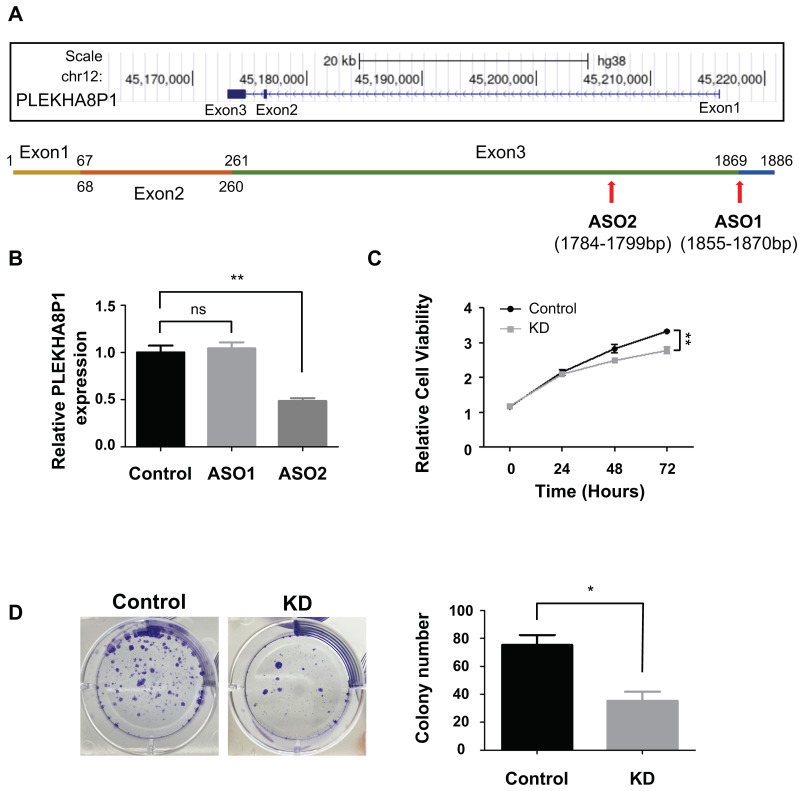
Downregulation of *PLEKHA8P1* inhibits proliferation of HCC cells. (**A**) The human *PLEKHA8P1* gene resides on chromosome 12 and consists of three exons. Antisense oligonucleotides were designed to target exon 3 (red arrows). (**B**) Knockdown efficiency was measured by qRT-PCR 48 h post-second transfection. Between two ASOs, ASO 2 significantly reduced the levels of *PLEKHA8P1*. (**C**) CCK-8 assays were performed to determine cell viability at indicated time-points (0, 24, 48, 72 h). (**D**) Representative images of colony formation assays. All data were acquired from three independent experiments and *p*-values were determined by two-tailed unpaired *t*-test analysis. * *p* < 0.05; ** *p* < 0.01; ns indicates no significance.

*PLEKHA8P1* is a transcribed processed pseudogene residing at 12q12 and consists of three exons (Figure 2A). To interrogate the potential significance of *PLEKHA8P1* in HCC cells, we conducted a loss-of-function study in FT3-7 using ASOs, which are 15–25 base-pair long single-stranded nucleic acids that mediate RNase-H degradation of target transcripts [38]. A pair of ASOs were designed against *PLEKHA8P1* (Figure 2A) and following transfection of ASOs into FT37 cells, qRT-PCR analysis indicated that ASO 2 showed greater efficacy in reducing *PLEKHA8P1* expression level relative to negative control (Figure 2B). Consequently, only ASO 2 was used in downstream perturbation experiments.

### 2.3. PLEKHA8P1 Promotes Proliferation in HCC Cells

**Figure 3:**
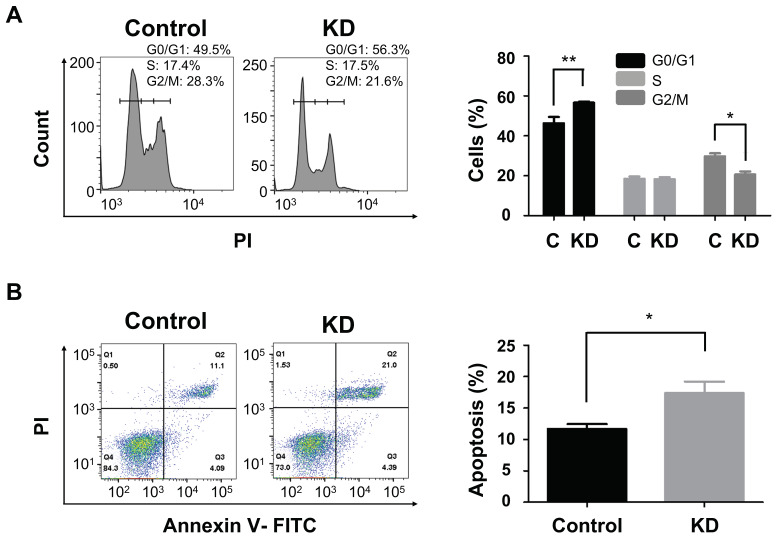
Downregulation of *PLEKHA8P1* promotes cell cycle arrest and apoptosis of HCC cells. (**A**) Analysis of cell cycle phase distribution through flow cytometry, with concurrent representation in histogram form. (**B**) Apoptotic cells were determined by flow cytometry and representative image of flow cytometry dot-plot is presented. All data were acquired from three independent experiments and *p*-values were determined by two-tailed unpaired t-test analysis. * *p* < 0.05; ** *p* < 0.01; C, control.

With *PLEKHA8P1* knockdown, we showed that cancer cell proliferation (Figure 2C) and its related colony formation properties (Figure 2D) are inhibited. Cell viability as scored by CCK8 assay is lower while fewer colonies are observed in *PLEKHA8P1* KD groups relative to negative control. Since *PLEKHA8P1* is purported to promote cell proliferation in HCC cell line which is attributable to dysregulated cell cycle and reduced apoptosis [14,15], we examined the effect of *PLEKHA8P1* silencing on both processes. Flow cytometry results showed cell cycle arrest at the G1/G0 phase in *PLEKHA8P1* KD group (Figure 3A). Moreover, we found that the percentage of PI^+^ Annexin V^+^ apoptotic cells increased in *PLEKHA8P1* KD compared to negative control (Figure 3B), suggesting that decreased cell viability in *PLEKHA8P1* KD is due to induction of cell cycle arrest and increased apoptosis. We were thus able to demonstrate that *PLEKHA8P1* promotes cell proliferation of liver cancer cells through both cell cycle regulation and cell apoptosis.

### 2.4. PLEKHA8P1 Promotes Invasion and Migration in HCC Cells

**Figure 4:**
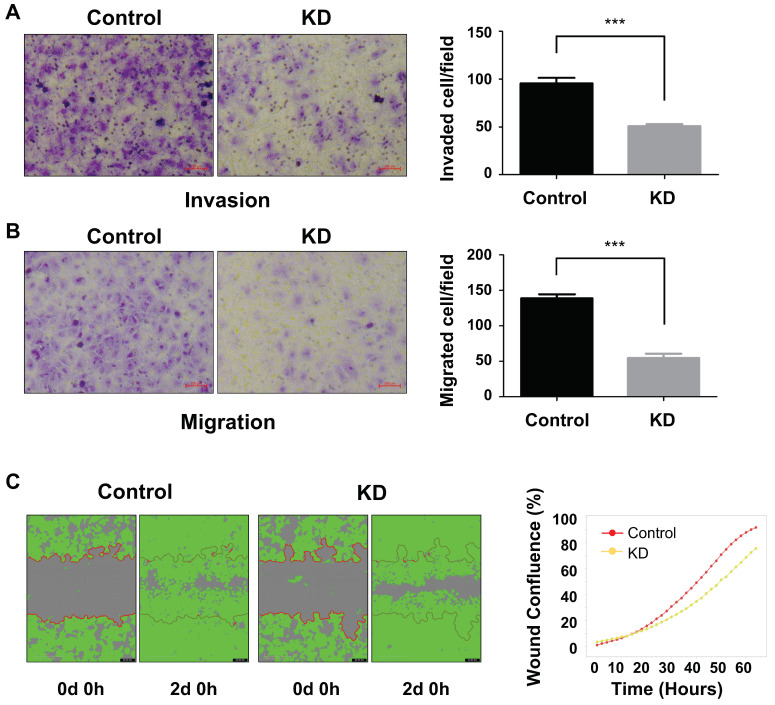
Downregulation of *PLEKHA8P1* inhibits migration and invasion of HCC cells. (**A**,**B**) Transwell assays were performed to determine invasive (**A**) and migratory (**B**) properties of cancer cells. Representative images of six random fields per well are shown (×200 magnification). Scale bars represent 100 μm. (**C**) Wound-healing assays were conducted for up to 3 days. Representative images of each group at specific time-points are presented. All data were acquired from three independent experiments and *p*-values were determined by two-tailed unpaired *t*-test analysis. *** *p* < 0.001.

Invasion and migration are essential steps in cancer metastasis [16,17] and we investigated whether these features are affected by *PLEKHA8P1* expression levels. Transwell assays showed that invasive and migratory abilities are reduced in *PLEKHA8P1* KD compared to control groups (Figure 4A,B). Similar results were obtained in wound-healing assays (Figure 4C), whereby cell migration to close the created wound was retarded upon *PLEKHA8P1* KD compared to control treatment.

Taken together, these results demonstrated that in addition to cell survival, *PLEKHA8P1* contributes to invasive and migratory abilities of liver cancer cells.

### 2.5. PLEKHA8P1 as a Positive Regulator of PLEKHA8

**Figure 5:**
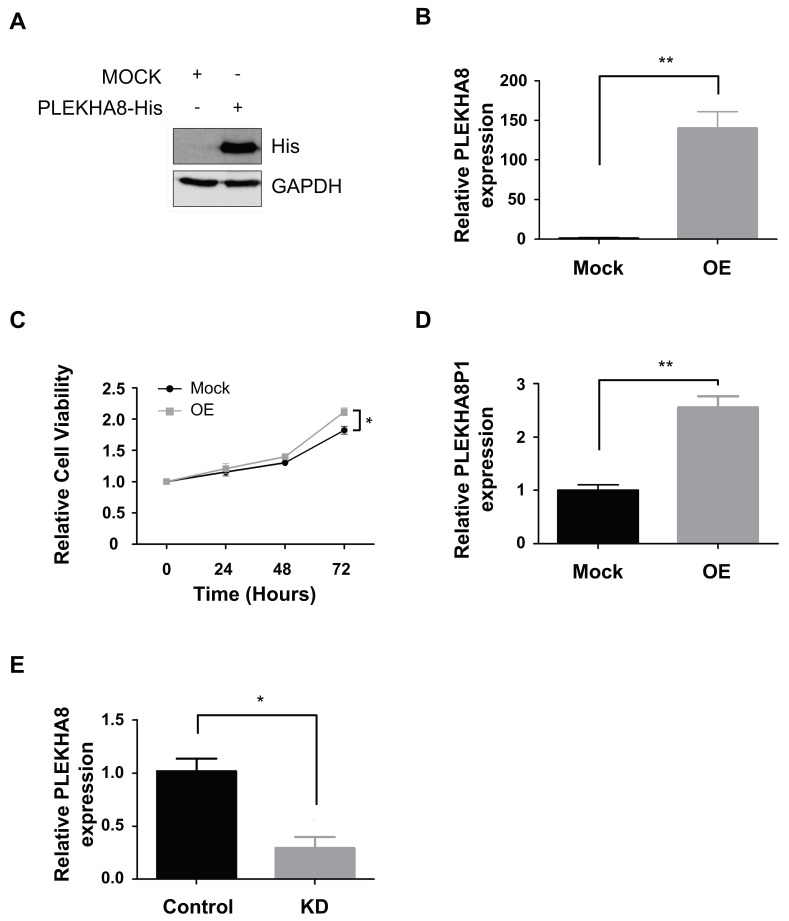
Overexpression of *PLEKHA8* promotes proliferation of liver cancer cells. (**A**) *PLEKHA8* mRNA level was assessed in *PLEKHA8P1* knockdown cells by RT-qPCR. (**B**) Overexpressed *PLEKHA8* was confirmed by Western blot analysis 48 h after transfection. (**C**) Overexpressed *PLEKHA8* was verified via RT-qPCR 48 h after transfection. (**D**) CCK-8 assays were performed to determine cell viability at indicated time-points (0, 24, 48, 72 h). (**E**) *PLEKHA8P1* expression level was assessed in *PLEKHA8* overexpressed cells by RT-qPCR. All data were acquired from three independent experiments and *p*-values were determined by two-tailed unpaired *t*-test analysis. * *p* < 0.05; ** *p* <0.01.

Studies have shown that the parental gene *PLEKHA8* plays an oncogenic role wherein it promotes cancer cell growth [18], while its knockdown displays opposite phenotypes [19]. In order to establish the relationship between the pseudogene-parent pair alongside functional interrogation of *PLEKHA8* in liver cancer cells, we constructed a *PLEKHA8*-overexpression plasmid. *PLEKHA8* was successfully overexpressed in FT3-7 as determined by Western blot and qRT-PCR (Figure 5A,B). We were able to confirm that *PLEKHA8* exhibits oncogenic features through cell survival analysis post-*PLEKHA8* overexpression (OE), whereby increased cancer cell proliferation is observed in *PLEKHA8*-OE groups (Figure 5C).

Though RNAseq expression data indicated that both *PLEKHA8P1* and *PLEKHA8* are up-regulated in tumor samples (Figure 1E), we used qRT-PCR to confirm in vitro the manner of correlation between their expression levels. qRT-PCR data generated in HCC cell line showed that *PLEKHA8P1* is up-regulated in *PLEKHA8* OE groups (Figure 5D) whilst *PLEKHA8* is reduced correspondingly in *PLEKHA8P1* KD groups (Figure 5E). Given this indication of positive correlation between *PLEKHA8P1* and *PLEKHA8*, we were able to reason that the pseudogene affects cancer cell growth by positive regulation over its parental gene, one which has been revealed to hold oncogenic properties.

### 2.6. PLEKHA8P1 Confers 5-FU Resistance to HCC Cells

5-FU is a well-utilized chemotherapeutic agent in several cancers [39,40,41,42], and is commonly used in combination with other drugs in TACE treatment [39,40]. However, drug resistance is a considerable impediment to effective cancer treatment with 5-FU [42], making the advancement of studies related to drug resistance in HCC a critical challenge to resolve.

**Figure 6:**
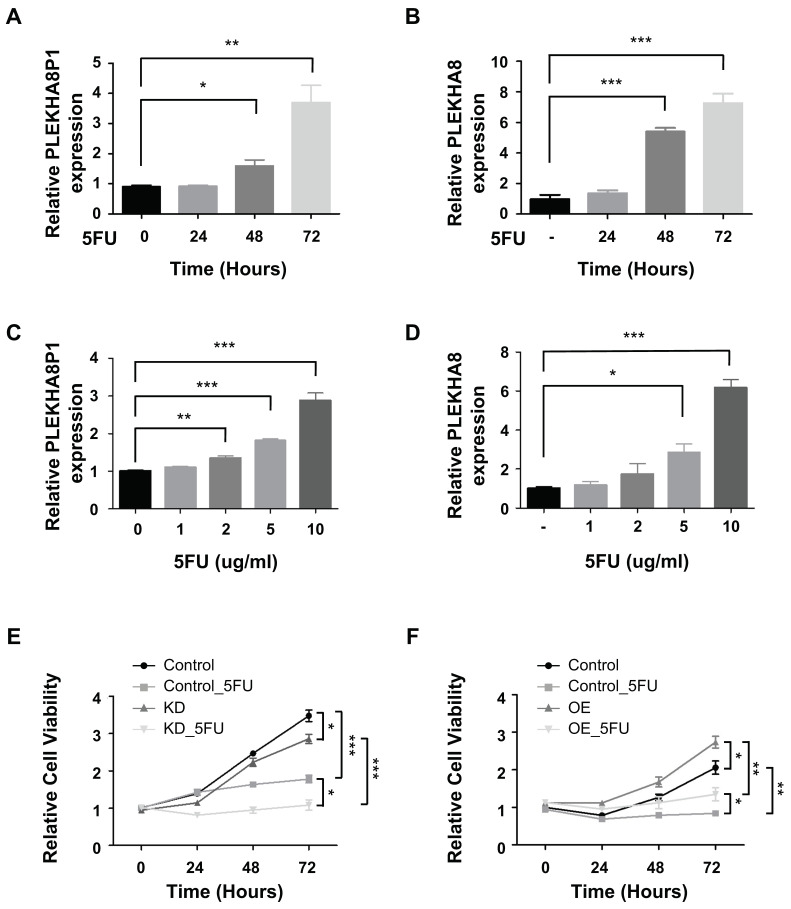
Downregulation of *PLEKHA8P1* sensitizes HCC cells to 5-FU. (**A**–**D**) qRT-PCR was carried out to determine the expression levels of *PLEKHA8P1* and *PLEKHA8* in 5-FU-treated cells. (**A**,**B**) Cells were treated with 5-FU (10 μg/mL) for the indicated time (0, 24, 48, 72 h). (**C**,**D**) Cells were treated with indicated concentrations (0, 1, 2, 5, 10 μg/mL) of 5-FU for 72 h. (**E**) FT3-7 cells were transfected with ASOs and cell viability assays were conducted in the presence or absence of 5-FU (10 μg/mL). (**F**) FT-3-7 cells were transfected with *PLEKHA8* plasmid and cell viability assays were conducted in the presence or absence of 5-FU (10 μg/mL). All data were acquired from three independent experiments and *p*-values were determined by two-tailed unpaired t-test analysis. * *p* < 0.05; ** *p* < 0.01; *** *p* < 0.001.

We observed expression levels of both pseudogene *PLEKHA8P1* and parental gene *PLEKHA8* in 5-FU-treated cells in a time- (Figure 6A,B) and dose-(Figure 6C,D)dependent manner. Since a corresponding increase in gene expression level is associated with elevation in amount and intensity of the cytotoxic drug, we reasoned that both *PLEKHA8P1* and *PLEKHA8* confer 5-FU resistance in HCC cells. Thus, we explored if knockdown of the pseudogene increases chemosensitivity of liver cancer to 5-FU, and found that 5-FU-mediated cytotoxicity is significantly increased in *PLEKHA8P1* K/D (Figure 6E). Similarly, overexpression of PLEKHA8 led to improved cell survival (Figure 6F), providing another line of evidence that modulation of both *PLEKHA8P1* and *PLEKHA8* alters the cytotoxic effect of 5-FU in HCC.

Our results thus demonstrate that a *PLEKHA8P1-PLEKHA8* axis could exert a cytoprotective effect against 5-FU, thus connoting both molecules as novel therapeutic targets in HCC-related chemoresistance studies.

## 3. Discussion

Treatment of hepatocellular carcinoma (HCC), which forms the bulk of primary liver cancer, is dependent on both diagnosis stage and underlying condition of patients, and most are recommended for transarterial chemoembolization (TACE) or systemic treatment. However, drug resistance impedes chemotherapy-based treatment, resulting in the majority of patients presenting with disease progression. Coupled with difficulties in early diagnosis that could enable curative therapies, the range of effective therapies for HCC remains limited. Though several protein-coding genes and long non-coding RNAs (lncRNAs) that confer chemoresistance to drugs such as 5-FU have been identified, the critical need for more precise identification and characterization remains given the poor 5-year survival rate.

It is now widely accepted that the vast majority of the human genome is pervasively transcribed into lncRNAs, with numbers and functionalities in various layers of gene regulation growing by the day. Investigations have revealed several lncRNAs to be regulators of processes that drive chemoresistance, including cell survival, migration/invasion and epithelial-to-mesenchymal transition. A relatively new class of lncRNAs—pseudogene-derived lncRNAs—is also widely implicated in tumorigenesis, and work by Poliseno et al. on pseudogene (PTENp1) and its parental gene (tumor suppressor PTEN) characterized the former’s miRNA decoy function, initiating much interest in the competing endogenous RNA (ceRNA) mechanism of pseudogene-mediated gene regulation. This model, in which pseudogenes positively regulate their parental genes and unrelated genes via competitive binding to shared microRNAs (miRNAs), has since then been established as a major mechanism of pseudogene-derived lncRNAs in human cancer regulation.

In our present study, we sought to identify pseudogene candidates with known parental genes by first examining their differential gene expression. Out of the resulting 465 significantly dysregulated genes, we examined their prognostic values through Kaplan–Meier survival analyses (overall survival and disease-free progression) and correlation with histologic tumor grade. Ten pseudogenes displayed significance in all three analysis sets, and we selected the pseudogene *PLEKHA8P1* for further experimental validation, as it is reported to hold prognostic potential in both renal cell carcinoma [37] and colon cancer [43]. In addition, its parental gene *PLEKHA8* has been previously characterized as on oncogene, and our analysis reflects the possibility, since *PLEKHA8* is up-regulated in tumor samples and also predicts poor prognosis for high-expression patients. We then characterized the role of *PLEKHA8P1* through antisense-oligonucleotide-mediated knock-down studies, and showed that loss of *PLEKHA8P1* resulted in reduced proliferation, invasion, and migration of HCC cell line derivative FT3-7. It would have been better to use additional HCC cell lines to support our findings, but we took a recent analysis that presented *PLEKHA8P1* as a significant risk factor for survival rate in colon cancer patients [43] as support for our hypothesis. We also noted with considerable interest that *PLEKHA8* parental gene promotes tumor progression, as observed in colon cancer and HCC cell lines (HepG2, MHCC97H, SNU-449) and xenograft tumor models [34,35,36], by enhancing Wnt/β-catenin signaling, one of the oncogenic pathways often targeted for HCC treatment [44]. On top of previous studies, our data demonstrate the oncogenic capabilities of both *PLEKHA8P1* and *PLEKHA8*.

A positive correlation in expression level between PLEKHA8P1 and the above reported oncogenic parental gene in both a time and dose-dependent manner suggested a possible ceRNA regulatory mechanism between the two counterparts. Furthermore, we identified shared miRNA binding sites corresponding to mir-516-b and mir-4766-5p, both tumor suppressors in various cancers (data not shown). As our interest lay in examining effect of the lncRNA-parental gene pair on chemoresistance, we explored such potential in the context of 5-FU treatment in HCC. *PLEKHA8P1* KD and *PLEKHA8* overexpression individually confers chemoresistance to 5-FU, as made apparent by the decreased and increased cell survival relative to control, respectively. Our data are thus indicative of a *PLEKHA8P1*-*PLEKHA8* axis in modulating chemoresistance, with *PLEKHA8P1* as a possible ceRNA for miRNAs that inhibit *PLEKHA8*, which in turn promotes tumor progression through Wnt/β-catenin signaling.

As our study is a snapshot of the role of identified pseudogene-lncRNA in relation to its parental gene in both tumorigenesis and 5-FU-mediated chemoresistance, we anticipate that further studies would be required to corroborate our current findings. Some immediate examples include: a more thorough perturbation technique, such as CRISPR/Cas9 knock-out of genes, to fully interrogate the extent of loss-of-function phenotype, as our data recorded a knockdown rate of 40–50%; use of miRNA mimics/inhibitors to validate the possible role of pseudogene as a ceRNA; rescue assays examining the *PLEKHA8P1*-*PLEKHA8* axis directly; and xenograft studies to confirm the tumorigenic role of *PLEKHA8P1*.

Nonetheless, our study revealed the novel role of *PLEKHA8P1* in HCC cell line as an oncogenic pseudogene-lncRNA and confirmed cancer-related functions of its parental gene *PLEKHA8*. Our data suggest that *PLEKHA8P1* positively regulates *PLEKHA8* as a miRNA sponge, and alteration of *PLEKHA8P1* expression affects chemosensitivity of HCC cell line FT3-7 to 5-FU, highlighting the potential of *PLEKHA8P1* as a novel therapeutic target in chemoresistance in liver cancer.

## 4. Methods and Materials

### 4.1. Raw Data Acquisition

Transcriptome data of TCGA-LIHC were retrieved from Genomic Data Commons (GDC) Data Portal [45] using the TCGAbiolinks R package [46]. We downloaded HTseq-Count data of 418 samples including 369 primary tumor and 49 solid tissue normal samples. Additional clinical data were obtained from the cBioPortal [47].

### 4.2. Differential Gene Expression Analysis

Differentially expressed gene analysis was performed using the DESeq2 R package [48]. All 421 HTseq-count files were used to generate a DESeqDataSet by using the DESeqDataSetFromHTSeqCount() function and genes with less than a total of 10 were filtered out. Differential expression analysis was conducted by using a single function, DESeq(). |Log_2_ Fold Change| ≥ 0.5, and adjusted *p*-values < 0.05 were used as criteria for defining statistically significant differentially expressed genes (DEGs). A principal component analysis (PCA) plot was generated by using the plotPCA (returnData = TRUE to customize the plot). A volcano plot to visualize the negative binomial test result was generated with EnhancedVolcano R package [49]. Count data were variance stabilizing transformed by the vsd() function of DESeq2. A total of 418 samples were unsupervised hierarchical clustered by the ward.D2 linkage method [50] and visualized by using the pheatmap R package [51].

### 4.3. Correlation Analysis

The correlation between histological tumor grade and pseudogenes was investigated in 364 samples with pertaining “Neoplasm Histologic Grade” information. Neoplasm Histologic grade is constituted with Grade 1 to Grade 4. For the correlation analysis, Grade 1 and 2 were grouped into Group1 and Grade 3 and 4 into Group2. Spearman correlation test was conducted between the grouped neoplasm histologic grade and each normalized counts of pseudogenes using cor.test(method = ’spearman’) function in R.

### 4.4. Survival Analysis

Clinical information (overall survival month, disease free month) from 368 and 317 TCGA-LIHC primary tumor samples were obtained from the cBioPortal, respectively. For all differentially expressed pseudogenes, Kaplan–Meier curve plotting and the log-rank test were performed using survival [52,53,54] and survminer [55] R packages. Samples were divided into two groups by a median expression value of each gene. The *p*-values of multiple tests were merged by Fisher’s method [56] implemented with metap R package [57]. 

### 4.5. Cell Culture and Transfection

FT3-7 cells were cultured in DMEM (Hyclone, Logan, UT, USA) supplemented with 10% fetal bovine serum (FBS; Hyclone, Logan, UT, USA), Penicillin-Streptomycin (Gibco, Waltham, MA, USA) in an incubator containing 5% CO_2_ at 37 °C. Cells were treated with 5-fluorouracil (5-FU) (Sigma, St. Louis, MO, USA) at indicated doses and time. Antisense oligonucleotides (ASOs) used to knockdown *PLEKHA8P1* gene were designed and purchased alongside negative control ASOs from Qiagen. 5 × 10^5^ cells were seeded in 6-well plates and ASOs (20 nM) were transfected into cells with Lipofectamine RNAiMax (Invitrogen, Waltham, MA, USA) as per manufacturer’s instructions. A second transfection was conducted after 24 h to increase knockdown efficiency. The ASO sequences are as follows: 

Negative control ASO: 5′-AACACGTCTATACGC-3′,

*PLEKHA8P1* ASO1: 5′-TTGCTGTGAAATCATG-3′,

*PLEKHA8P1* ASO2: 5′-ACACTTTAGCACTTTA-3′.

For *PLEKHA8* gene overexpression, *PLEKHA8* sequence was cloned into pcDNA3.1 V5/His A vector (Invitrogen, Waltham, MA, USA). pcDNA3.1 *PLEKHA8* V5/His A plasmid (4 μg, 6-well scale) was transfected into cells with Lipofectamine 2000 (Invitrogen, Waltham, MA, USA) as per manufacturer’s instructions. 

### 4.6. Cell Viability Assay

Cell viability was measured using Cell Counting Kit-8 (CCK-8; Dojindo, Kumamoto, Japan) as per manufacturer’s protocol. Briefly, transfected cells were seeded in 96-well plates and 10 μL of CCK-8 reagent was added to each well at stipulated time-points (0, 24, 48 and 72 h). After incubation at 37 °C for 1 h, absorbance at 450 nm was measured in a microplate reader (Molecular Devices, San Jose, CA, USA).

### 4.7. Colony Formation Assay

Following transfection, 2 × 10^3^ cells were seeded in 6-well plates and cultured for 12 days. Cells were fixed using 4% paraformaldehyde and stained with 1% crystal violet for colony enumeration.

### 4.8. Flow Cytometry for Apoptosis

Apoptotic cells were detected using FITC Annexin V Apoptosis Detection Kit I (556547, BD, Franklin Lakes, NJ, USA) in accordance with manufacturer’s instructions. In brief, harvested cells were washed with cold PBS and resuspended in 1× binding buffer. A total of 5 µL of FITC annexin V and 5 µL propidium iodide (PI) were added and following 15 min of incubation at room temperature in dark condition, cells were analyzed by flow cytometer (FACS Canto II, BD, USA).

### 4.9. Cell Cycle Analysis

For cell cycle analysis, harvested cells were fixed with 75% ethanol at −20 °C for 24 h. After washing twice with 1 × PBS, cells were incubated with PI and 500 μg/mL RNase A (Invitrogen, Waltham, MA, USA) for 15 min at room temperature in the dark. Each cell phase was assessed based on DNA content via flow cytometer (FACS Canto II, BD, USA).

### 4.10. Invasion and Migration Assays

24-well transwell plates (8 μm pore size, 3422, Corning Inc., Corning, NY, USA) were used for invasion and migration assays. For invasion assays, transwell inserts were coated with Matrigel (354234) diluted in serum-free medium. A total of 3 × 10^4^ cells resuspended in serum-free medium were seeded in the inserts, while 600 μL of medium containing 10% FBS was added into the lower chamber. After 24–48 h, invaded cells were fixed with 4% paraformaldehyde and stained with 1% crystal violet. For migration assays, the lower side of the insert was coated with gelatin (G1393, Sigma) diluted in PBS. A total of 1.5 × 10^4^ cells resuspended in serum-free medium were seeded in inserts while 600 μL of medium containing 10% FBS was added to the lower chamber. Following 24–48 h, migrated cells were fixed with 4% paraformaldehyde and stained with 1% crystal violet. Six random fields per well were photographed by microscopy (Carl Zeiss, Jena, Germany) and counted.

### 4.11. Wound-Healing Assay

Transfected cells were seeded in 96-well ImageLock plate (4379, Sartorius, Gottingen, Germany) at a density of 3 × 10^4^ cells/well. Wounds were created by IncuCyte WoundMaker Tool (4563, Sartorius, Germany) and cell debris were removed by washing with 1 × PBS. Cells were monitored and analyzed by IncuCyte Live-Cell analysis System (Sartorius, Germany) for up to 3 days.

### 4.12. RNA Isolation and Quantitative Real-Time PCR

Total RNA was extracted from cultured cells with TRIzol reagent (Invitrogen, USA) and reverse transcriptase PCR (RT-PCR) was conducted with 2 μg of RNA. qRT-PCR was carried out using SYBR Green dye (Invitrogen) and detected by LightCycler480 II (LC480; Roche, Basel, Switzerland) with the following cycling conditions: pre-incubation at 95 °C for 5 min, followed by 45 cycles of amplification at 95 °C for 10 s, 60 °C for 10 s, and 72 °C for 10 se. The relative gene expression levels were normalized to *GAPDH* and calculated by the 2^−ΔΔCp^ method. The primer sequences used for qRT-PCR are as follows: 

*GAPDH*: Forward, 5′-AATCCCATCACCATCTTCCA-3′, 

*GAPDH*: Reverse, 5′-TGGACTCCACGA CGTACTCA-3′,

*PLEKHA8*: Forward, 5′-AGCGACTGAAGCCCTCTTGT-3′

*PLEKHA8*: Reverse, 5′-TTTCTGTTGGATTTCTTAGGGCTG-3′

*PLEKHA8P1*: Forward, 5′-CAGCCTTTACCTCCCTGCCA-3′,

*PLEKHA8P1*: Reverse, 5′-TGCCCAGCAGCCATCATACA-3′.

### 4.13. Western Blot Analysis

Total protein was extracted from cultured cells with RIPA lysis buffer (CST, Danvers, MA, USA) containing protease inhibitor cocktail (Bio-Rad, Hercules, CA, USA). A total of 30 μg of protein lysate was separated in SDS-PAGE gels and transferred onto 0.22 μm nitrocellulose membranes (GenDEPOT, Katy, TX, USA). Membranes were blocked with 5% skim milk and incubated overnight at 4 °C with indicated primary antibodies: anti-GAPDH (sc-25778, 1:1000 dilution, Santa Cruz, Dallas, TX, USA), anti-His (ab9108, 1:1000 dilution, Abcam, Cambridge, UK), followed by incubation with rabbit HRP-conjugated secondary antibody (31460, Thermo Scientific, Waltham, MA, USA). Signals were detected by ECL solution (RPN2232, GE Healthcare, Chicago, IL, USA).

## 5. Conclusions

In this study, we have characterized a selected pseudogene-derived lncRNA *PLEKHA8P1* that could function as a prognostic marker, promotes tumor progression and affects 5-FU-mediated chemoresistance through positive regulation of its parental gene *PLEKHA8.* Given our initial observations and known role of parental gene *PLEKHA8* in tumorigenesis, we highlight the potential of *PLEKHA8P1* as a novel therapeutic target in chemoresistance in liver cancer.

## Data Availability

Transcriptome data of TCGA-LIHC were retrieved from Genomic Data Commons (GDC) Data Portal [45] while additional clinical data were obtained from cBioPortal [47]. All data generated and used in this study are available for anyone to utilize upon reasonable request.

## Supplementary Materials

The following are available online at https://www.mdpi.com/article/10.3390/ijms22147614/s1. Table S1. PLEKHA8P1_ST S1, Table S2. PLEKHA8P1_ST S2, Table S3. PLEKHA8P1_ST S3.
